# Biphasic dysglycemia induced by pentamidine and detected by intermittently scanned continuous glucose monitoring: a case report of a possible drug-drug interaction with clopidogrel and literature review

**DOI:** 10.1186/s40780-026-00549-3

**Published:** 2026-02-07

**Authors:** Yuki Nakano, Noriko Kuhara, Rintaro Sogawa, Hisanari Yasukochi, Yusuke Okayama, Misato Motoya, Hidehiro Ishii, Hirotsugu Hasuwa, Chisato Shimanoe

**Affiliations:** 1 Department of Pharmacy, Saga University Hospital, Saga, Japan; 2 Department of Pharmacy, Saiseikai Futsukaichi Hospital, Fukuoka, Japan; 3 Department of Respiratory Medicine, Saiseikai Futsukaichi Hospital, Fukuoka, Japan; 4 Department of Diabetes and Endocrinology, Saiseikai Futsukaichi Hospital, Fukuoka, Japan

**Keywords:** Pentamidine, Hypoglycemia, Hyperglycemia, Glycemic variability, IsCGM, Drug-induced dysglycemia

## Abstract

**Background:**

Pentamidine is an alternative treatment for Pneumocystis pneumonia (PCP) in patients who are intolerant to sulfamethoxazole-trimethoprim; however, its use is limited by serious adverse effects, including glycemic fluctuations. Herein, we report a case in which intermittently scanned continuous glucose monitoring (isCGM) was used to monitor real-time glycemic fluctuations during pentamidine therapy, enabling successful completion of treatment. To contextualize this case, we also conducted a targeted literature review of pentamidine-induced glucose dysregulation.

**Case presentation:**

An 86-year-old Japanese man undergoing immunosuppressive therapy for rheumatoid arthritis was diagnosed with PCP and initially treated with sulfamethoxazole-trimethoprim. Because of suspected adverse effects of sulfamethoxazole-trimethoprim, the patient was switched to intravenous pentamidine as second-line therapy. Given the risks associated with advanced age, abnormal kidney function, and concomitant use of clopidogrel, an organic cation transporter 1 (OCT1) inhibitor that may affect pentamidine disposition, glycemic monitoring was initiated using isCGM. The patient experienced nocturnal hypoglycemia soon after initiating pentamidine treatment, which persisted after pentamidine discontinuation. He subsequently developed marked hyperglycemia associated with impaired endogenous insulin secretion, necessitating temporary regular insulin administration and sitagliptin combination therapy. His glycemic control gradually stabilized, and recovery of pancreatic β-cell function was confirmed by increased urinary C-peptide levels. In a literature review of 83 reports published between 1995 and 2025, only nine cases of pentamidine-associated dysglycemia were identified, and this was the only case that demonstrated biphasic glucose dysregulation with both hypoglycemia and hyperglycemia.

**Conclusion:**

This case demonstrates that biphasic glucose dysregulation associated with pentamidine (early hypoglycemia followed by delayed-onset hyperglycemia) can be captured using isCGM. Real-time glucose monitoring during pentamidine therapy enabled early intervention and safe completion of treatment. Glycemic monitoring using isCGM may be beneficial in patients receiving pentamidine, especially those with abnormal kidney function, older patients, or those receiving concomitant OCT1 inhibitor therapy.

## Introduction

Pentamidine is an antiprotozoal agent that has long been used to treat infections including *Pneumocystis* pneumonia (PCP), leishmaniasis, and African trypanosomiasis [1, 2]. Among these, PCP is currently the most common indication in clinical practice. PCP is a life-threatening opportunistic infection that occurs in immunosuppressed patients, including those with human immunodeficiency virus infection and those on immunosuppressive therapy such as corticosteroids, and has a reported mortality rate of 25.4% [3]. The first-line treatment for PCP is high-dose sulfamethoxazole-trimethoprim, but its use is limited by adverse effects such as abnormal kidney function and electrolyte disturbances [4, 5]. Alternative agents for the treatment of PCP include intravenous pentamidine, atovaquone, and the combination of clindamycin and primaquine, with pentamidine, and clindamycin-primaquine combination therapy recommended as second-line options for treating mild to severe cases [6]. However, in Japan, pentamidine and atovaquone, but not primaquine, are currently approved for clinical use.

Pentamidine is associated with several adverse effects, including nephrotoxicity, hypotension, arrhythmias, and glycemic fluctuations [7]. Both hypoglycemia and hyperglycemia have been reported as manifestations of glycemic fluctuations [8], and can necessitate treatment discontinuation and adversely affect prognosis. Therefore, close monitoring of glycemic fluctuations is essential during pentamidine therapy to ensure early detection and timely intervention. Particular caution should be exercised in older patients, especially those receiving concomitant medications that may lead to clinically significant drug-drug interactions.

Intermittently scanned continuous glucose monitoring (isCGM) is a minimally invasive device that allows real-time tracking of interstitial glucose levels [9]. Since its clinical introduction in Europe in 2014, isCGM has become widely used, particularly in patients with diabetes mellitus [10]. The device continuously measures interstitial glucose via a subcutaneous sensor and provides current glucose levels, trends, and historical data by scanning the sensor with a reader or smartphone, thereby minimizing the need for frequent capillary blood sampling [9, 10]. Although isCGM offers practical advantages in monitoring glycemic variability, its approved indications vary by country, subject to national regulatory differences, and in Japan its approval is currently limited to diabetes mellitus.

Herein, we report a case in which pentamidine was administered as an alternative to sulfamethoxazole-trimethoprim in an immunosuppressed patient of advanced age, and isCGM was used to monitor real-time glycemic fractions throughout the treatment course.

## Case presentation

An 86-year-old Japanese man (height: 144 cm; body weight: 45.6 kg; body mass index: 21.8 kg/m^2^) was admitted to our hospital for evaluation of progressive respiratory symptoms. His medical history included rheumatoid arthritis (treated with prednisolone 10 mg/day and tacrolimus 1.5 mg/day), bronchial asthma (treated with montelukast 10 mg/day, sustained-release theophylline 200 mg/day, and fluticasone furoate/vilanterol 200 μg/40 μg combination inhaler), prior cerebral infarction (treated with clopidogrel 75 mg/day), and lumbar spinal canal stenosis (treated with celecoxib 200 mg/day). Other supportive medications included lansoprazole 15 mg/day, alfacalcidol 1 μg/day, magnesium oxide 660 mg/day, epinastine 20 mg/day, and ferric citrate 100 mg/day.

**Fig. 1:**
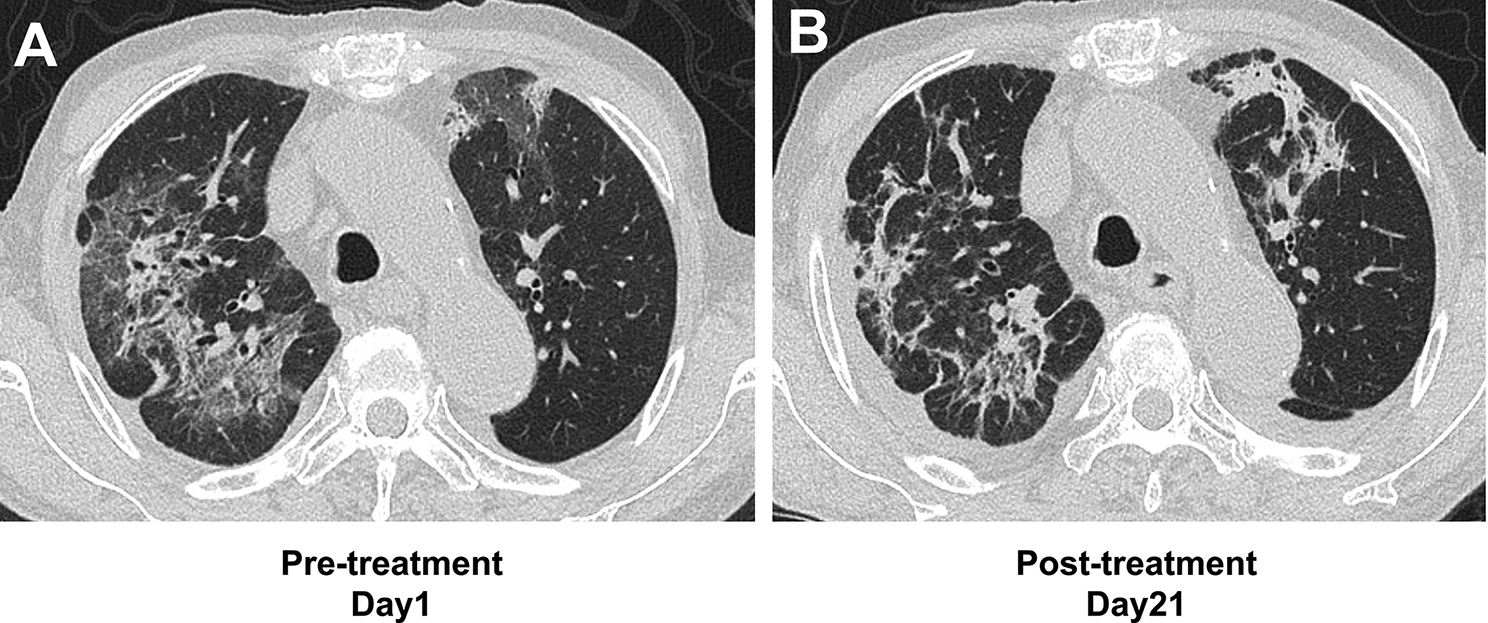
Chest computed tomography (CT) findings before and after treatment. (**A**) Pre-treatment (day 1): CT shows bilateral ground-glass opacities. (**B**) Post-treatment (day 21): following treatment with sulfamethoxazole-trimethoprim and pentamidine, the opacities have improved, with residual post-inflammatory changes

**Fig. 2:**
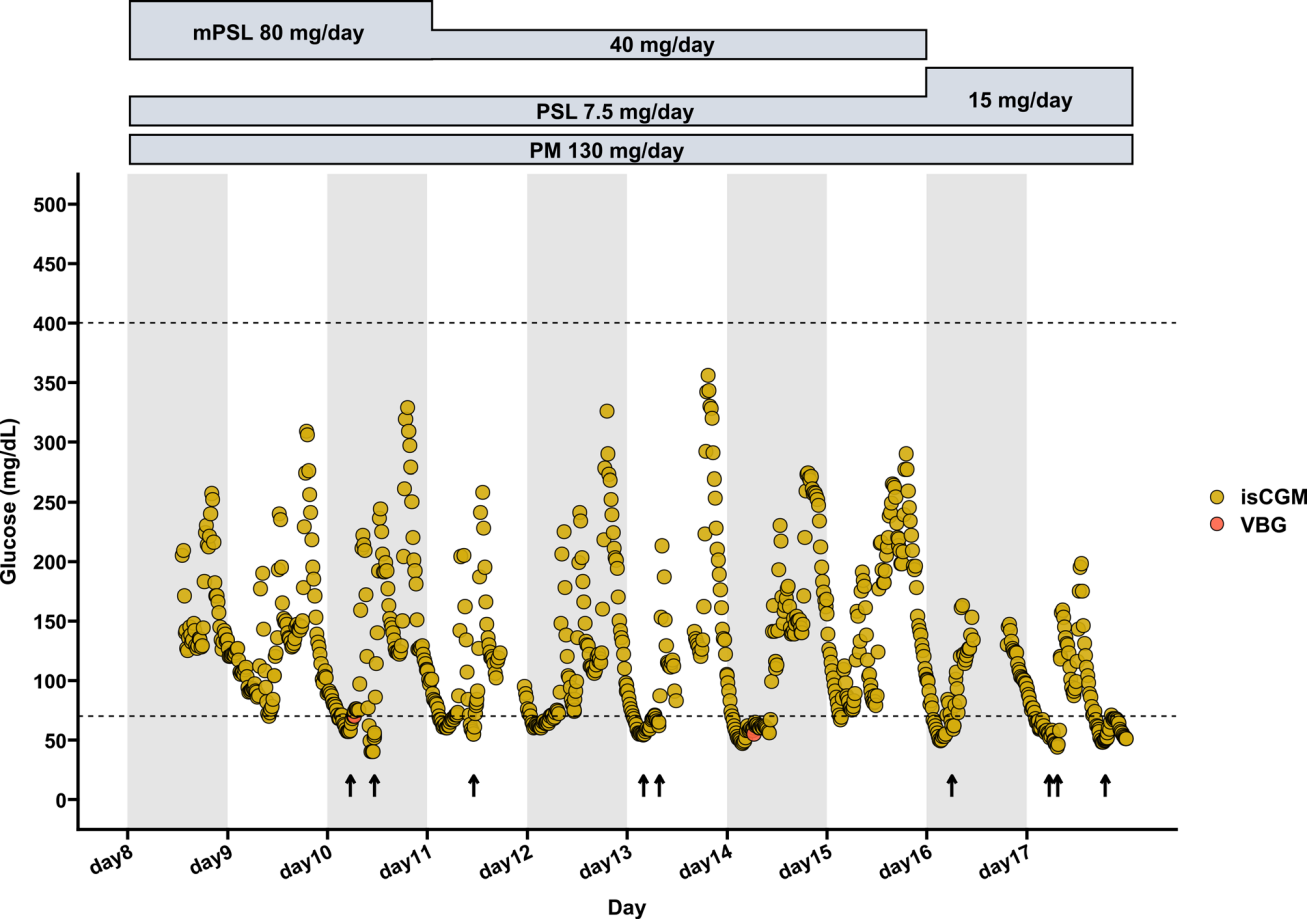
Time course of glucose concentrations during pentamidine treatment. The X-axis represents days, and the Y-axis represents glucose concentration (mg/dL). The dashed lines indicate the thresholds for hypoglycemia (70 mg/dL) and hyperglycemia (400 mg/dL). The arrow indicates the timepoint at which oral administration of 10 g doses of glucose was initiated. Yellow circles represent interstitial glucose concentrations measured by isCGM, and red circles represent glucose concentrations measured by venous blood sampling. Abbreviation: isCGM, intermittently scanned continuous glucose monitoring; mPSL, methylprednisolone; PM, pentamidine; PSL, prednisolone; VBG, venous blood glucose

Chest computed tomography showed bilateral ground-glass opacities suggestive of *Pneumocystis* pneumonia (PCP), cytomegalovirus pneumonia, or an exacerbation of interstitial lung disease (Fig. 1A). The patient was diagnosed with PCP based on an increased β-D-glucan level (55.2 pg/mL; normal < 20 pg/mL) and a positive polymerase chain reaction result for *Pneumocystis jirovecii* DNA in bronchoalveolar lavage fluid. His C-reactive protein (CRP) level was also elevated (8.23 mg/dL; normal < 0.14 mg/dL). Oral sulfamethoxazole-trimethoprim (3,600 mg-720 mg/day) was initiated as first-line therapy for the treatment of PCP. Intravenous methylprednisolone (mPSL) 80 mg/day was initiated on day 5 of treatment owing to progression of respiratory failure, manifesting as a PaO_2_/FiO_2_ ratio of 196 calculated from SpO_2_ using the Severinghaus equation. Sulfamethoxazole-trimethoprim was discontinued on day 7 owing to abnormal kidney function, hyperkalemia, and gastrointestinal adverse effects including vomiting, which were attributed to sulfamethoxazole-trimethoprim. Intravenous pentamidine (130 mg/day) was initiated on day 8 as second-line therapy, based on a creatinine clearance (Ccr) of 23.6 mL/min at initiation, which was reduced from a baseline level of approximately 35–40 mL/min calculated using the Cockcroft–Gault formula. Given the potential risk of developing glucose dysregulation associated with pentamidine, isCGM (FreeStyle Libre; Abbott Diabetes Care, Alameda, CA, USA) was implemented based on the advice of an endocrinologist, in accordance with institutional ethical guidelines and with approval from the institutional review board. Frequent episodes of nocturnal hypoglycemia were observed from day 10. Each episode was managed with 10 g of oral glucose (Fig. 2). Pentamidine was discontinued on day 17 following clinical improvement, as indicated by a reduction in the CRP level to 1.46 mg/dL. By this time, the patient’s kidney function had improved, with the Ccr increasing to 34.9 mL/min. Because of the continued use of prednisolone for rheumatoid arthritis, prophylactic sulfamethoxazole-trimethoprim (400 mg/80 mg once daily, three times per week) was initiated. Imaging on day 21 demonstrated radiological resolution with residual post-inflammatory organizing changes (Fig. 1B).

**Fig. 3:**
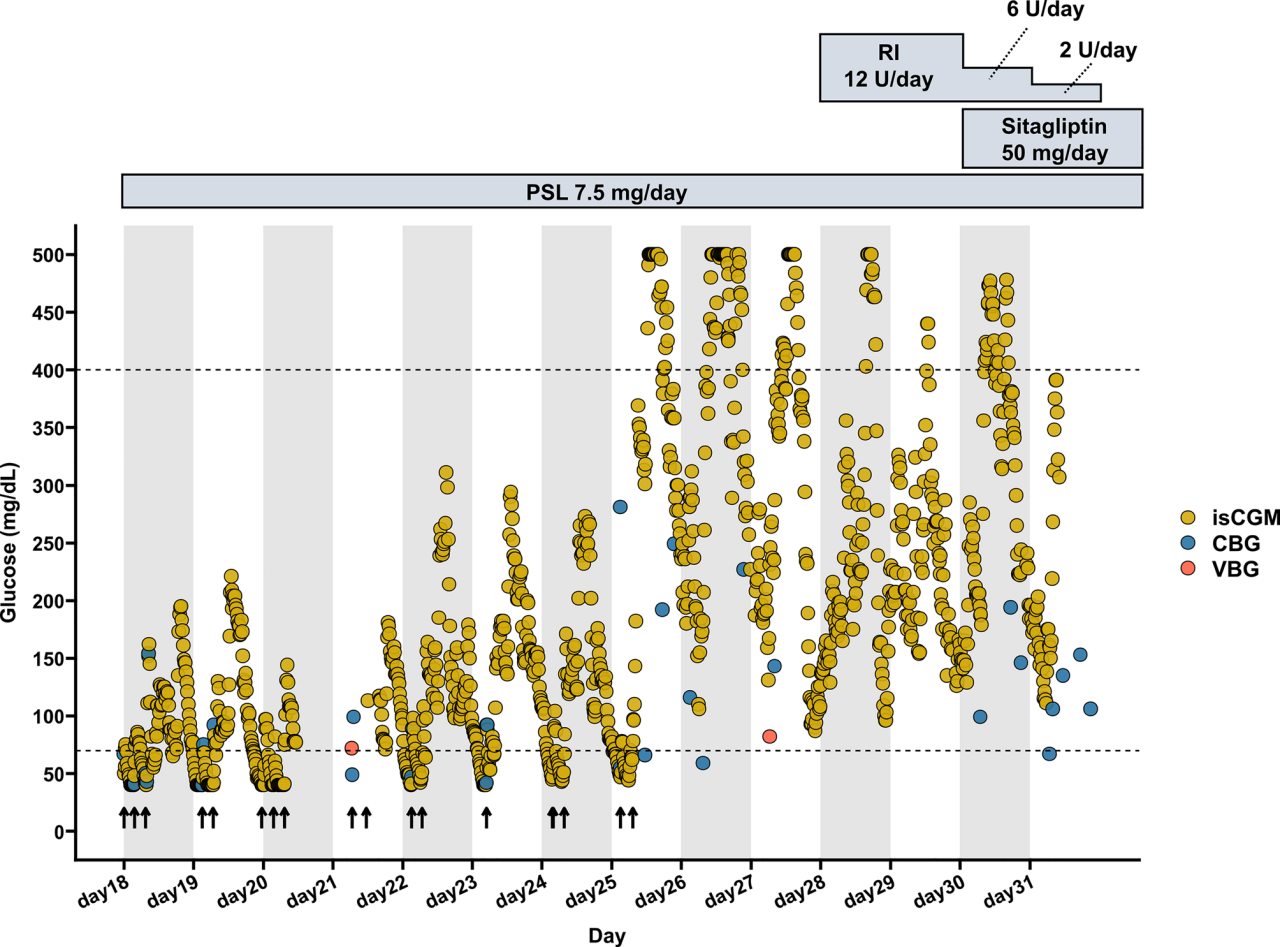
Time course of glucose concentrations after pentamidine discontinuation. The X-axis represents days, and the Y-axis represents glucose concentration (mg/dL). The dashed lines indicate the thresholds for hypoglycemia (70 mg/dL) and hyperglycemia (400 mg/dL). The arrow indicates the timepoint at which oral administration of 10 g doses of glucose was initiated. Yellow circles represent interstitial glucose concentrations measured by isCGM, blue circles represent capillary blood glucose concentrations measured by point-of-care testing, and red circles represent glucose concentrations measured by venous blood sampling. Abbreviation: CBG, capillary blood glucose; isCGM, intermittently scanned continuous glucose monitoring; PSL, prednisolone; RI, regular insulin; VBG, venous blood glucose

After discontinuation of pentamidine, glucose monitoring using isCGM was continued. The frequent episodes of nocturnal hypoglycemia persisted even after pentamidine was discontinued, necessitating repeated oral glucose administration. Unexpectedly, from day 25, the patient developed sustained hyperglycemia, with interstitial glucose levels exceeding the upper detection limit of the device (≥500 mg/dL) (Fig. 3). On day 26, the patient’s urinary C-peptide excretion was 5.6 μg/day, indicating markedly reduced endogenous insulin secretion. Consequently, sliding-scale insulin therapy was started on day 28, and sitagliptin 50 mg/day was added on day 30. The antidiabetic agents were withdrawn as endogenous insulin secretion recovered. Regular insulin administration was discontinued on day 31, and sitagliptin was discontinued on day 33. Continuous glucose monitoring using isCGM was discontinued on day 31, and subsequent capillary blood glucose monitoring showed no clinically relevant fluctuations. On day 42, reassessment of insulin secretory capacity showed that urinary C-peptide excretion had increased to 27.6 μg/day, indicating recovery of β-cell function. The patient was transferred to a rehabilitation facility on day 50 in a stable condition.

## Literature review

**Fig. 4:**
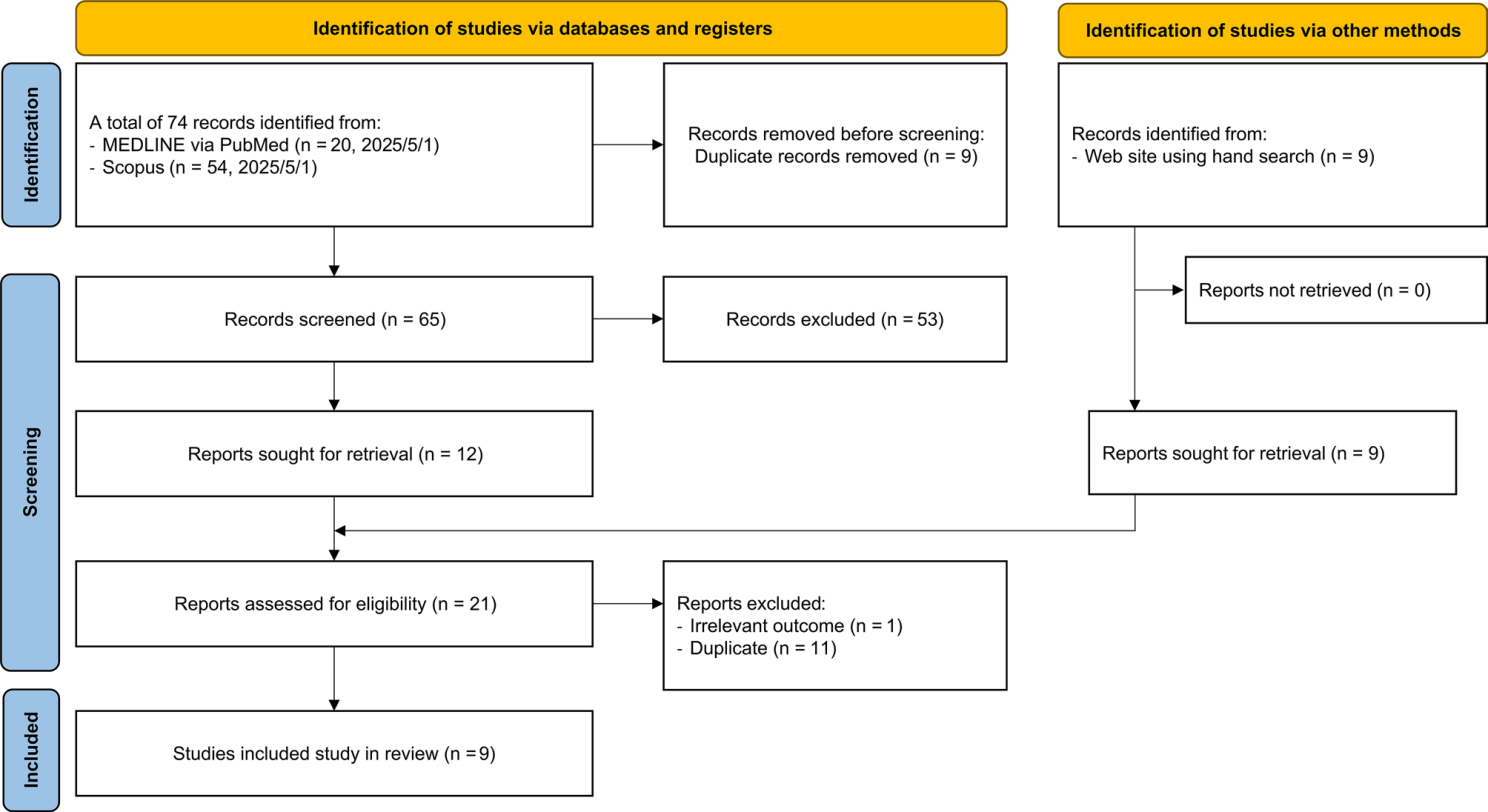
PRISMA flow diagram for literature selection. Abbreviations: PRISMA, preferred reporting items for systematic reviews and meta-analyses

To contextualize this case, we conducted a targeted review of reports on pentamidine-induced glucose dysregulation published between1995 and 2025. An electronic literature search was performed in May 2025 using MEDLINE via PubMed, Scopus, and manual web-based searches. The detailed search strategy and search terms are provided in Table 1. We selected this 30-year timeframe to account for historical changes in pentamidine salt formulations, which may influence its pharmacokinetic and toxicological profiles. The review focused on identifying individual case reports or case series describing hypoglycemia, hyperglycemia, or biphasic glucose abnormalities associated with pentamidine therapy. All titles and abstracts were independently screened by two reviewers (Y.N. and H.Y.). Full texts of potentially relevant articles were retrieved and assessed for eligibility. Disagreements between reviewers were resolved through discussion and consensus. This review was conducted in accordance with the Preferred Reporting Items for Systematic Reviews and Meta-Analyses (PRISMA) guidelines [11]. A PRISMA flow diagram summarizing the screening and selection process is provided in Fig. 4.

**Table 1:** Search terms for the systematic review

Search number	MEDLINE via PubMed	Scopus
S1	pentamidine”[All Fields] OR “pentamidine isethionate”[All Fields] OR “pentamidine”[MeSH Terms]	TITLE-ABS-KEY(pentamidine OR “pentamidine isethionate”)
S2	“hyperglycemia”[All Fields] OR “hypoglycemia”[All Fields] OR “glucose intolerance”[All Fields] OR “insulin resistance”[All Fields] OR “diabetes mellitus”[All Fields] OR “blood glucose”[All Fields] OR “glycemic control”[All Fields] OR “glucose metabolism”[All Fields] OR “glucose homeostasis”[All Fields] OR “hyperglycemia”[MeSH Terms] OR “hypoglycemia”[MeSH Terms] OR “diabetes mellitus”[MeSH Terms]	TITLE-ABS-KEY(“hyperglycemia” OR “hypoglycemia” OR “glucose intolerance” OR “insulin resistance” OR “diabetes mellitus” OR “blood glucose” OR “glycemic control” OR “glucose metabolism” OR “glucose homeostasis”
S3	“Case Reports”[Publication Type]	TITLE-ABS-KEY(“case report” OR “case series”)
S4	“1995/01/01”[Date - Publication]: “2025/5/1”[Date - Publication]	PUBYEAR > 1994 AND PUBYEAR < 2026
Total	S1 AND S2 AND S3 AND S4	S1 AND S2 AND S3 AND S4

Abbreviation: MEDLINE, Medical Literature Analysis and Retrieval System Online

A total of 83 records were identified: 20 from MEDLINE via PubMed, 54 from Scopus, and 9 through manual web-based searches. Screening resulted in 9 cases being included in the review [12–20] (Table 2). Unlike our patient, none of the 9 cases identified in the literature review had both hypoglycemia and hyperglycemia. Among the included case, 7 of the 9 cases were in individuals with human immunodeficiency virus (HIV) infection. Abnormal kidney function was present in 4 patients at the time of pentamidine administration. isGCM was used to monitor blood glucose levels in only 1 of the 9 published case reports. Of the patients who developed dysglycemia, two ultimately progressed to insulin-dependent diabetes mellitus, and one to non-insulin-dependent diabetes mellitus without recovery of glucose tolerance.

**Table 2:** Summary of reported cases of pentamidine-induced glucose dysregulation from 1995 to 2025

Case	Age (years)	Sex	Underlying disease	HIV status	Daily pentamidine dose	Glucose metabolism disorder	isGCM monitoring	TTO (days)	Pancreatitis onset	Diabetes outcome	Pre-existing DM	Pre-existing abnormal kidney function	Reference
1	75	F	PCP	+	4 mg/kg/day	Hypoglycemia	No	10	No	―	No	No	[12]
2	58	M	PCP	−	4.46 mg/kg/day	Hypoglycemia	Yes	9	No	―	Yes	No	[13]
3	42	M	PCP	+	240 mg/body/day	Hyperglycemia	No	3	No	Recovery	No	No	[14]
4	26	F	Leishmaniasis	−	4 mg/kg/day	Hyperglycemia	No	14	No	IDDM	No	Yes	[15]
5	29	M	PCP	+	3 mg/kg/day	Hyperglycemia	No	11	No	Death	No	No	[16]
6	29	M	PCP	+	4.35 mg/kg/day	Hyperglycemia	No	27	Yes	IDDM	No	No	[17]
7	33	M	Rhinocerebral mucormycosis	+	Unknown	Hyperglycemia	No	20	No	Death	No	Yes	[18]
8	27	M	PCP	+	4 mg/kg/day	Hyperglycemia	No	21	Yes	NIDDM	No	Yes	[19]
9	29	M	PCP	+	4 mg/kg/day	Hyperglycemia	No	39	No	Recovery	No	Unknown	[20]
Present case	87	M	PCP	−	2.85 mg/kg/day	Hypo- and hyperglycemia	Yes	3/18	No	Recovery	No	Yes	This article

Abbreviations: +, positive; −, negative; DM, diabetes mellitus; F, female; HIV, isCGM, intermittently scanned continuous glucose monitoring; human immunodeficiency virus; IDDM, insulin-dependent diabetes mellitus; M, Man; NIDDM, non-insulin-dependent diabetes mellitus; PCP, *Pneumocystis* pneumonia; TTO, time to onset

## Discussion

To our knowledge, this is the first report to dynamically capture biphasic glucose dysregulation associated with pentamidine using isCGM in real time, along with measuring corresponding changes in insulin secretory capacity using urinary C-peptide. In this case, isCGM enabled the detection of both early nocturnal hypoglycemia and delayed-onset, sustained hyperglycemia even after pentamidine discontinuation. These real-time observations provided actionable insights that guided timely glucose supplementation and insulin initiation, thereby contributing to the safe completion of pentamidine therapy. This case provides unique pathophysiological evidence for a temporal shift from insulin overproduction to β-cell depletion and demonstrates the potentially useful role of isCGM for improving the safety of pentamidine treatment in high-risk patients.

Pentamidine-induced glucose dysregulation has been attributed to pancreatic β-cell injury [21]. This injury leads to excessive insulin release and consequent hypoglycemia. With continued insulin secretion, intracellular insulin stores become depleted, which can result in a transition to hyperglycemia. This biphasic glycemic pattern—initial hypoglycemia followed by subsequent hyperglycemia—has been reported previously [22]. Notably, in this case, this dynamic change was visualized continuously in real time using isCGM.

In our patient, real-time monitoring of interstitial glucose variability using isCGM documented these fluctuations. Continuous glucose monitoring with isCGM enabled the identification of a transition to hyperglycemia on day 8 following a 10-day course of pentamidine, which was consistent with the time to onset reported previously (median 19.3 days) (Table 2). Although insulin therapy was initiated in response to hyperglycemia, it was later discontinued following the recovery of endogenous insulin secretion (as indicated by changes in the urinary C-peptide excretion from 5.6 to 27.6 μg/day). This observation supports the hypothesis that insulin depletion contributes to hyperglycemia in pentamidine-induced β-cell injury. Pentamidine-induced β-cell dysfunction may be at least partially reversible, provided that permanent β-cell injury has not occurred [14, 20]. However, given that many of the patients in the previously reported cases progressed to insulin-dependent diabetes mellitus, caution is warranted when administering pentamidine because of the potential for long-term β-cell injury induced by pentamidine.

Consideration of kidney function and the concomitant medication use was crucial when interpreting the development of pentamidine-induced dysglycemia in this case. Pentamidine-induced toxicity has been reported to be associated with use of high doses, repeated courses of pentamidine therapy, or accumulation due to abnormal kidney function [22]. In our literature review, 70% (7 of the 10) of the cases, including our patient, had at least one risk factor, including advanced age, prolonged exposure (≥2 weeks), or abnormal kidney function, with abnormal kidney function being the most frequent risk factor (40%, 4 of the 10). In contrast, in the only previously reported case in which isCGM was used, none of these high-risk factors were present [13]. In that case, reported by Hayakawa et al. [13], steroid-induced diabetes mellitus was present as an underlying condition, and isCGM was introduced primarily for routine glycemic management of pre-existing diabetes rather than to anticipate pentamidine-induced glucose dysregulation. Although the pentamidine dose in our patient was adjusted according to kidney function, symptomatic hypoglycemia occurred in the early morning of day 3. Continuous monitoring with isCGM likely enabled the detection of this early-onset hypoglycemia, which is consistent with previous reports [13]. Pentamidine-induced hypoglycemia frequently occurs at night or in the early morning when intermittent blood glucose measurements are impractical. Real-time isCGM enables continuous monitoring and early detection of unrecognized hypoglycemia, allowing timely glucose correction and safer management of high-risk patients. At the same time, circadian rhythm-related fluctuations in glucose levels may have been further influenced by concomitant medications. High-dose mPSL administered during the daytime may have produced a hyperglycemic effect that temporarily masked the clinical manifestation of β-cell toxicity [23, 24]. Nevertheless, the early occurrence of hypoglycemia suggests that pentamidine-induced β-cell injury developed sooner than expected and supports the clinical utility of isCGM for early detection and safe management of pentamidine-associated dysglycemia. In patients with abnormal kidney function, continuous glucose monitoring may be particularly useful for detecting unpredictable glycemic fluctuations following pentamidine administration.

Another potential contributor to altered glucose homeostasis in this case is the co-administration of clopidogrel, which is metabolized to clopidogrel carboxylate, a potent inhibitor of organic cation transporter 1 (OCT1) in vitro. This metabolite may have affected the pharmacokinetics of pentamidine, leading to elevated plasma concentrations and prolonged systemic exposure [25]. Pentamidine has a large volume of distribution (3 L/kg) [26] and is rapidly cleared from plasma after administration due to its uptake and accumulation in tissues such as the liver and kidneys, primarily via OCT1, whereas renal excretion accounts for only approximately 5% of its total clearance [27]. Consequently, its apparent clearance is generally high (248 ± 91 L/h) [28], and plasma concentrations typically decrease below the detection limit within a few hours, although pharmacological activity persists in tissues. OCT1 inhibition by clopidogrel carboxylate may have inhibited transporter-mediated uptake of pentamidine into organs involved in its elimination, resulting in a functional reduction in clearance and increased systemic exposure. Although screening thresholds for transporter-mediated drug-drug interactions have been proposed for many transporters, no threshold is available for OCT1 [29, 30]. However, consistent with this interpretation, cimetidine, an OCT1 inhibitor with a well-established interaction profile, has been shown to reduce the clearance of pentamidine when co-administered with pentamidine [31]. In addition, the anatomical and physiological characteristics of the pancreas may also contribute to pentamidine distribution. The pancreas expresses organic cation transporters (OCTs) at low levels but has highly permeable capillaries, which allow drugs to accumulate in the interstitial space. Based on available pharmacokinetic and mechanistic evidence, the drug-drug interaction caused by co-administration of clopidogrel may have facilitated OCT-independent off-target accumulation of pentamidine in pancreatic tissue and contributed to the early β-cell toxicity observed in this case. In our patient, avoiding concomitant OCT1 inhibitor use might have reduced the risk of pentamidine-induced glucose dysregulation. Switching from clopidogrel to prasugrel, which has a low potential for transporter-mediated interactions, was appropriate in our patient, given the approved indication [32].

This case report has several limitations. First, neither plasma nor tissue concentrations of pentamidine and clopidogrel were measured directly; therefore, the pharmacokinetic considerations that we present are speculative, based on previously published reports and theoretical assumptions. Second, other factors such as age-related alterations in metabolic function may have contributed to the observed glucose dysregulation. Third, although isCGM was clinically useful in capturing glycemic variability, discrepancies may be present between interstitial and plasma glucose levels, particularly during periods of rapid fluctuation. Fourth, genetic polymorphisms of OCT1 were not assessed in this case, and the potential influence of individual variability in transporter function remains unknown [33]. However, genetic polymorphisms of OCT1 vary substantially among ethnic groups, and few variants associated with reduced OCT1 activity have been reported in the Japanese population. Last, this report describes a single case and therefore does not allow for definitive conclusions regarding causality. Despite these limitations, this case highlights the clinical utility of isCGM in capturing both early-onset hypoglycemia during pentamidine administration and persistent hyperglycemia following its discontinuation. The temporal association with clopidogrel co-administration suggests a possible drug-drug interaction, which warrants further investigation.

In summary, biphasic glucose dysregulation induced by pentamidine may present as initial hypoglycemia followed by subsequent hyperglycemia after treatment cessation, demonstrating the importance of continuous glucose monitoring. Real-time glucose monitoring using isCGM may be useful in high-risk patients, such as those with abnormal kidney function, advanced age, or receiving concomitant OCT1 inhibitors.

## Data Availability

The data supporting the findings of this case are available from the corresponding author on reasonable request.

## Declarations

### Compliance with reporting guidelines

This report was prepared in accordance with the CARE (CAse REports) guidelines.

## Abbreviations

CRP: C-reactive protein
isCGM: Intermittently scanned continuous glucose monitoring
mPSL: Methylprednisolone
OCT1: Organic cation transporter 1
PCP: *Pneumocystis* pneumonia
